# The Sole *Mycobacterium smegmatis* MazF Toxin Targets tRNA^Lys^ to Impart Highly Selective, Codon-Dependent Proteome Reprogramming

**DOI:** 10.3389/fgene.2019.01356

**Published:** 2020-02-14

**Authors:** Valdir Cristovao Barth, Nancy A. Woychik

**Affiliations:** ^1^ Department of Biochemistry and Molecular Biology, Robert Wood Johnson Medical School, Rutgers University, Piscataway, NJ, United States; ^2^ Rutgers Cancer Institute of New Jersey, New Brunswick, NJ, United States

**Keywords:** MazF, toxin, antitoxin, transcriptome, ribosome, translation

## Abstract

Survival of mycobacteria, both free-living and host-dependent pathogenic species, is dependent on their ability to evade being killed by the stresses they routinely encounter. Toxin-antitoxin (TA) systems are unique to bacteria and archaea and are thought to function as stress survival proteins. Here, we study the activity of the endoribonuclease toxin derived from the MazEF TA system in *Mycobacterium smegmatis*, designated MazEF-ms. We first enlisted a specialized RNA-seq method, 5’ RNA-seq, to identify the primary RNA target(s) of the MazF-ms toxin. Just two tRNA species, tRNA^Lys-UUU^ and tRNA^Lys-CUU^, were targeted for cleavage by MazF-ms at a single site within their anticodon sequence (UU↓U and CU↓U) to render these tRNAs nonfunctional for protein synthesis. The 5’ RNA-seq dataset also revealed hallmarks of ribosome stalling predominantly at Lys AAA codons even though both Lys tRNAs were cleaved by MazF-ms. Stalled ribosomes were then cleaved on their 5’ side by one or more RNases, resulting in very selective degradation of only those mRNAs harboring ribosomes stalled at Lys codons. This highly surgical, codon-dependent degradation of mRNA transcripts was validated using quantitative mass spectrometry of proteins that were newly synthesized during MazF-ms expression. The *M. smegmatis* proteome was altered as predicted, Lys AAA codon-rich proteins was downregulated while Lys AAA codon deficient proteins were upregulated. Analysis of specific subsets of proteins that were upregulated or downregulated was consistent with the growth-arrested phenotype of MazF-ms expressing cells. Curiously, the tRNA target and mechanism of action of MazF-ms paralleled that of one atypical MazF toxin in *M. tuberculosis*, suggesting manipulation of the levels of lysine tRNAs as the preferred conduit for reprogramming the proteomes *via* ribosome stalling at rare AAA codons in these GC-rich mycobacteria.

## Introduction

Stress is a constant threat to the survival of free-living organisms. Toxin-antitoxin (TA) systems are believed to act as one line of defense enlisted by free-living bacteria to survive the constant barrage of environmental assaults in their native habitats (reviewed in Yamaguchi et al., 2011; Harms et al., 2018). TA systems are operons comprising adjacent genes encoding two small (~10 kDa) proteins, an antitoxin and its cognate toxin. Based on current models developed through studies of *Escherichia coli* TA systems (reviewed in Yamaguchi et al., 2011; Harms et al., 2018), in the absence of stress the intrinsic activity of the toxin is sequestered by the formation of a stable TA protein-protein complex. However, in response to one or more specific stress triggers, the antitoxin is degraded by proteases. The resulting paucity of antitoxin results in an excess of free toxin which then acts on its specific intracellular target. Toxin action on its target(s) generally results in growth arrest which is characteristically reversible when the stress is released, enabling replenishment of antitoxin. Thus, TA systems appear to work well for pulses of stress instead of prolonged stress where a major physiological and structural transformation is warranted, i.e., conversion from vegetative state to a nearly dormant spore.

*Mycobacterium smegmatis* is most recognized as a laboratory surrogate for the study of features it shares with its pathogenic relative, *M. tuberculosis*. However, in nature *M. smegmatis* is a saprophyte that lives in ever changing environments within soil, water and on plants. Thus, it should be genetically hardwired for stress survival even though its genome harbors only a small fraction of TA systems compared to *M. tuberculosis* [three in *M. smegmatis* (Frampton et al., 2012) vs. ~90 in *M. tuberculosis* (Ramage et al., 2009; Sala et al., 2014)]. In this study, we determine the intracellular target and study the function of one of the three distinct TA system family toxins in *M. smegmatis*, the sole MazF family member that we designate as MazF-ms (MSMEG_4448). We found that the primary RNA targeted for cleavage by this endoribonuclease toxin was tRNA^LysUUU^, identical to one of the 11 MazF toxins in *M. tuberculosis,* MazF-mt9 (Schifano et al., 2016; Barth et al., 2019). We then document a spectrum of downstream events that lead to surgical remodeling of the proteome, which closely paralleled that which occur in *M. tuberculosis* (Barth et al., 2019). This striking conservation of tRNA targets underscores the importance of the relatively rare Lys AAA mRNA codon as an efficient conduit for modulating the physiology in both mycobacterial species through activation of MazF toxins.

## Materials and Methods

### Strains, Plasmids, and Reagents

All experiments were performed using *Mycobacterium smegmatis* strain mc^2^ 155 (ATCC 700084). *M. smegmatis* cells were grown at 37°C in Difco Middlebrook 7H9 media (BD) supplemented with 5 g/L albumin, 2 g/L dextrose, 0.085 g/L NaCl, 0.05% Tween 80 and 25 µg/ml kanamycin (for plasmid selection), under constant shaking at 200 rpm.

The gene MSMEG_4448, here referred as MazF-ms, was amplified by PCR from *M. smegmatis* genomic DNA using the oligos NWO2791 (5’-AGA TAC ATA TGC GGC GCG GCG ATA TCT ACA CCG CGG-3’) and NWO2792 (5’-AGA TAA AGC TTC ACC CGG CGA TTC CCA GAA AAA CC-3’). The amplified DNA was cloned into the anhydrotetracycline (ATC)-inducible plasmid pMC1s (Ehrt et al., 2005) modified to substitute unique NdeI-HindIII sites in place of ClaI-EcoRI to enable insertion of a gene with 5’NdeI-3’HindIII sites. MazF-ms expression was induced by adding ATC to the media at a final concentration of 200 ng/ml when cells reached an OD (600 nm) between 0.1 and 0.2 and compared to uninduced (-ATC) samples.

### RNA Isolation

In order to extract total RNA, ~50 ml of *M. smegmatis* cells were collected by centrifugation at 2000 g at 4°C for 5 min. Cell pellets were resuspended in Tri reagent (Zymo Research) and transferred to 2 ml lysing kit tubes (Bertin Corp.) containing 0.1 mm glass beads. Cells lysis was performed on a Precellys Evolution homogenizer (Bertin Corp.) by three consecutive 30-s pulses at 9,000 rpm, with 1 min cooling periods on ice in between each cycle. The samples were centrifuged for 5 min at 14,000 rpm at 4°C, and RNA was isolated from the supernatant using the Direct-zol RNA Miniprep Plus extraction kit (Zymo Research). After isolation, the samples were treated with 1 U of Turbo DNase as an extra genomic DNA removal step, purified using the RNA Clean and Concentrator kit (Zymo Research) and eluted in 40 μl of RNase-free water. RNA concentration was measured in a BioSpectrometer (Eppendorf) with a µCuvette.

### 5′ RNA-Seq

5’ OH libraries were constructed as previously described (Schifano et al., 2014). Briefly, in order to remove 5’ monophosphate RNA species, three μg of the purified RNA from induced and uninduced cultures were digested with 1 U Terminator at 30°C for 1 h. After purification using the RNA Clean and Concentrator kit (Zymo Research), the samples were phosphorylated using 3 U of T4 PNK at 37°C for 1 h and re-purified with the same kit. 5’ adapter (5′-GUUCAGAGUUCUACAGUCCGACGAUCNNNNNN-3′) was ligated using 1 U of T4 RNA ligase 1 (New England Biolabs) at 16°C for approximately 18 h. In order to remove the remaining free adapters, the adapter-ligated RNAs were resolved on a 6% TBE-Urea PAGE gel, excised and precipitated in isopropanol at −20°C. The purified RNAs were used in a reverse transcription reaction using Superscript IV (Thermo Fisher) and the degenerate primer (5′-GCCTTGGCACCCGAGAATTCCANNNNNNNNN-3′). The resulting cDNA was loaded into a 10% TBE-Urea gel and fragments between 80 and 500 nts were excised and precipitated. The cDNA libraries were amplified in a PCR reaction with Phusion HF DNA Polymerase (Thermo Fisher). The primers used were RP1 (5′- AATGATACGGCGACCACCGAGATCTACACGTTCAGAGTTCTACAGTCCGA -3′) and RPIX (5′-CAAGCAGAAGACGGCATACGAGATNNNNNNGTGACTGGAGTTCCTTGGCACCCGAGAATTCCA-3′), where the N’s represent the individual Illumina barcodes for each library. The amplified libraries between 150–450 bp were gel purified and subjected to single-end sequencing in an Illumina HiSeq 2500 or HiSeq4000 sequencer.

Note that our 5’ RNA-seq method specifically selects for RNA molecules with 5’ hydroxyl ends created upon cleavage by MazF-ms. Based on the detailed schematic of our method in Schifano et al. (2016), if reverse transcriptase should pause at tRNA modifications, the truncated cDNA would not contain the complement to the 5’ adapter sequence that was exclusively ligated to RNAs containing a 5’-OH. Without the adapter sequence, truncated cDNAs would not be amplified by PCR nor could they be sequenced by the Illumina primer which is also complementary to the 5’ adapter.

The resulting FASTQ files had the adapter sequences and the first 6 nucleotides of the 5’ end trimmed using Trimmomatic (Bolger et al., 2014). Reads were trimmed to 20 nts and the ones containing fewer than 20 nucleotides were excluded. The remaining reads were mapped to *M. smegmatis* genome (NCBI accession: CP000480.1) using bowtie 1.2 applying the parameters –n 0–l20 (Langmead et al., 2009). Next, we calculated the number of reads that started at a given genome position for each nucleotide in the genome. Genomic positions with 0 counts received a pseudo count of 1 in the uninduced sample. The counts were normalized by sequencing depth, in reads per million (rpm) of mapped reads and the counts of the induced sample were divided by the uninduced control to generate a fold change. Unless otherwise stated, we only considered positions with at least 50 rpm and 5 rpm in the induced sample for tRNA and mRNA genes, respectively, and a fold change >10. Sequence and frequency logos were generated by kpLogo (Wu and Bartel, 2017). The fastq files were submitted to NCBI’s Sequence Read Archive (SRA), under BioProject number PRJNA564437.

### Labeling of Newly Synthesized Proteins and Proteomic Analysis

To identify and quantify which proteins are translated after induction of MazF-ms (MSMEG_4448), three biological replicates were grown to an OD_600_ between 0.1 and 0.2 and divided into induced (+ATC) and uninduced samples. In order to label newly synthesized proteins, the methionine analog azidohomoalanine (AHA, AnaSpec) was added to the media at 50 µM after 4.5 h of MazF-ms induction. After 1.5 h of incubation with AHA, the cells were pelleted by centrifugation at 2000 g at 4°C for 10 min and washed with PBS. The cells were resuspended in a 2% CHAPS, 8M Urea buffer and lysed as described in the “RNA isolation” section, using Precellys Evolution homogenizer (Bertin Corp). The cell lysate was centrifuged and the AHA-containing proteins in the supernatant were captured using Click-iT™ Protein Enrichment Kit (ThermoFisher) followed by in-column trypsin digestion.

Digests were analyzed in two separate runs and combined. The data was analyzed as described previously (Barth et al., 2019), considering proteins with ≥15 detected spectral counts. Q-values are calculated using the fdrtool package of Strimmer (Strimmer, 2008) with significant changes at or below a q-value of 0.05. The raw files were deposited in Mass Spectrometry Interactive Virtual Environment (MassIVE) repository (accession number: MSV000084300).

## Results

### MazF-ms Expression Arrests Growth in *M. smegmatis*

**Figure 1:**
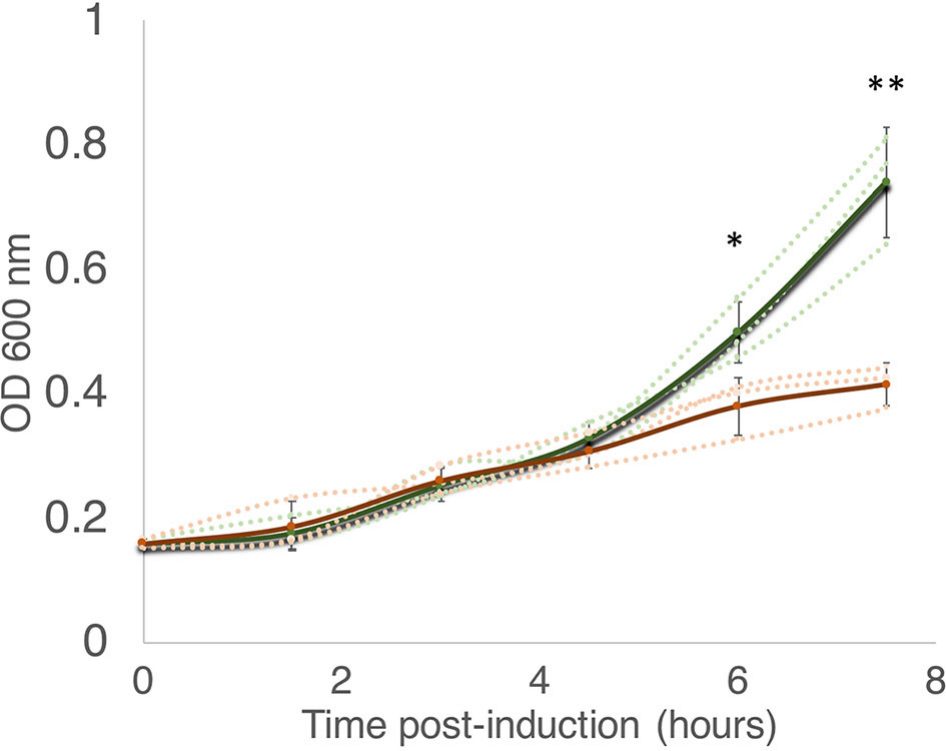
MazF-ms expression in *M. smegmatis* leads to growth inhibition. *M. smegmatis* cells harboring pMC1s-MazF-ms were grown in triplicate in supplemented 7H9 media until OD_600nm_ = ∼ 0.16. The cultures were split into induced (+ATC, orange lines) and uninduced (-ATC, green lines) and absorbance at 600 nm was determined every 1.5 h. Error bars represent the standard deviation from the average (solid line) of the three biological replicates (dotted lines). Asterisks represent statistical significance between induced and uninduced in a Student’s t test comparison (*, p-value = 0.011; **, p-value = 0.0046).

According to the Toxin-Antitoxin Database (TADB 2.0) (Xie et al., 2018), the *M. smegmatis* reference genome harbors only one gene from the MazF toxin family, annotated as MSMEG_4448 (here referred to as MazF-ms). To establish whether or not MazF-ms is toxic (i.e., leads to cell growth arrest) when expressed in *M. smegmatis*, we cloned the gene under the control of an ATC-inducible promoter in the pMC1s plasmid and transformed *M. smegmatis* mc^2^ 155 cells. The expression level from this plasmid is modest, a 4.5-fold induction, based on measurement of mCherry fluorescence (**Supplementary Figure 1**). MazF-ms expression led to pronounced growth arrest that started between 4.5 to 6 h (**Figure 1**) and was sustained for at least 18 h.

### MazF-ms Exclusively Targets Both tRNA^Lys^ Isoacceptors for Cleavage at a Single Site Within Their Anticodons

All members of the MazF family reported to date are single-strand, sequence-specific endoribonucleases. To help elucidate the molecular mechanism by which the ribonuclease activity of MazF-ms is able to regulate growth, we applied our specialized RNA seq method, 5’ RNA-seq, to find its RNA target(s) (Schifano et al., 2014). This technique was originally developed to selectively sequence transcripts based on their 5’ ends. Here, we apply 5’ RNA-seq to identify RNA fragments containing a 5’ hydroxyl (5’ OH) end, which are products of MazF toxin activity (Schifano et al., 2014). This approach also allows us to precisely map the cleavage position at a single nucleotide resolution.

**Figure 2:**
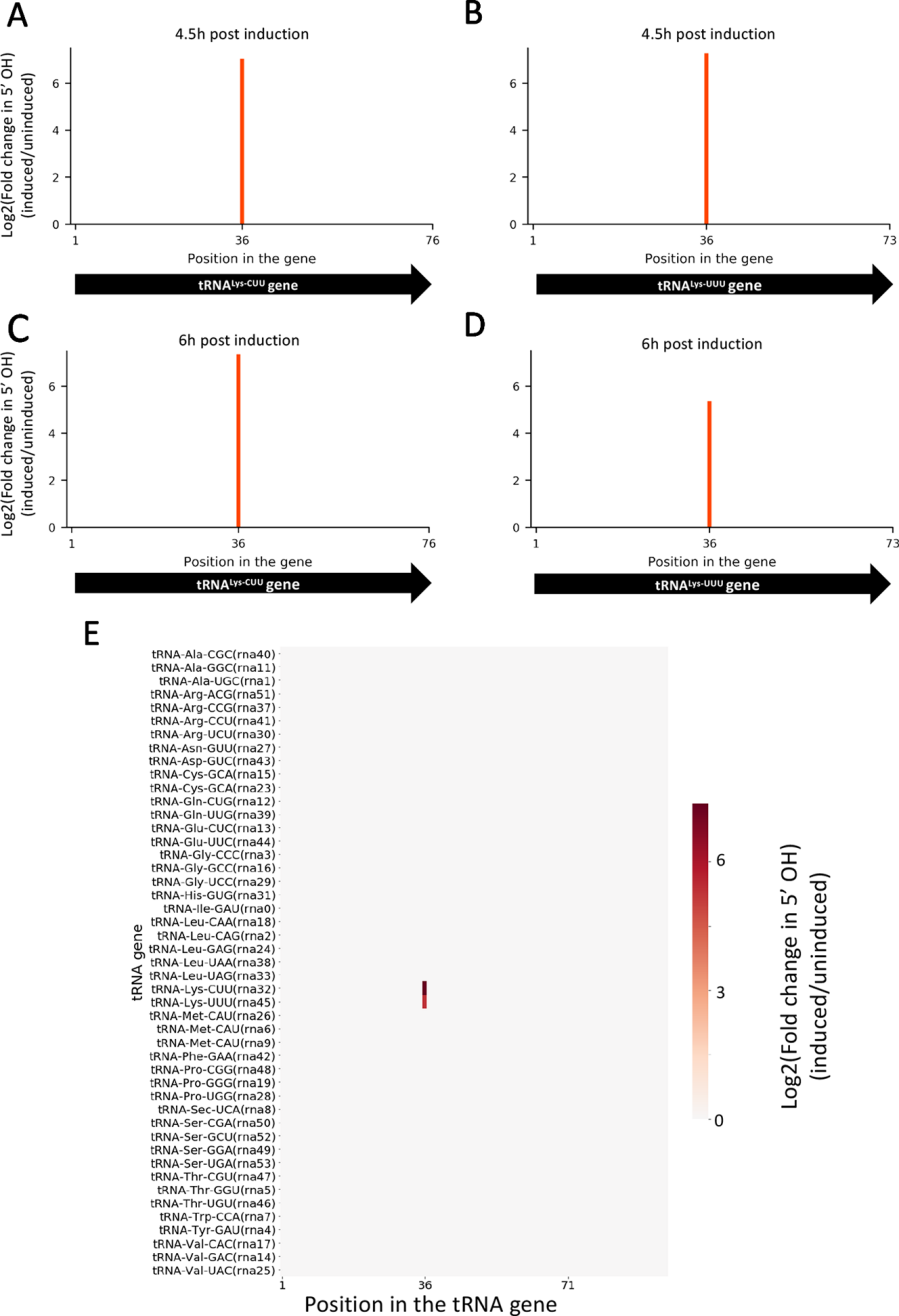
MazF-ms selectively targets both tRNA^Lys^ isoacceptors. **(A–D)** Fold changes of 5’OH (indicating endonucleolytic cleavage) within both tRNA^Lys-CUU^ and tRNA^Lys-UUU^ genes detected by 5’ RNA-seq. *M. smegmatis* cells expressing MazF-ms for 4.5 **(A, B)** or 6 h **(C, D)** were compared to uninduced controls. **(E)** Heatmap showing the fold change in 5’ OH levels in each position of all 47 *M. smegmatis* tRNA genes after 6 h of MazF-ms induction. The annotated tRNA gene ID (from genome CP000480.1) is shown in parentheses.

Given that the difference in growth between induced and uninduced cultures is more dramatically observed after 6 h post induction, we selected two time points for RNA isolation: one immediately before we observed growth separation (at 4.5 h) and one where the separation is significant (at 6 h). In both time points, 5’ RNA-seq identified internal cleavage of the only two tRNA^Lys^ isoacceptors [tRNA^Lys23-UUU^ and tRNA^Lys18-CUU^ (Lowe and Chan, 2016)] annotated in the *M. smegmatis* genome (**Figures 2A–D**). When compared to controls, MazF-ms-induced datasets showed an enrichment of 41 to 163-fold in intragenic 5’ OH ends at position 36 of both tRNA^Lys^ genes. There are 46 standard tRNAs in *M. smegmatis* and one selenocysteine tRNA, none of the other 45 tRNAs were cleaved by MazF-ms (**Figure 2E**).

**Figure 3:**
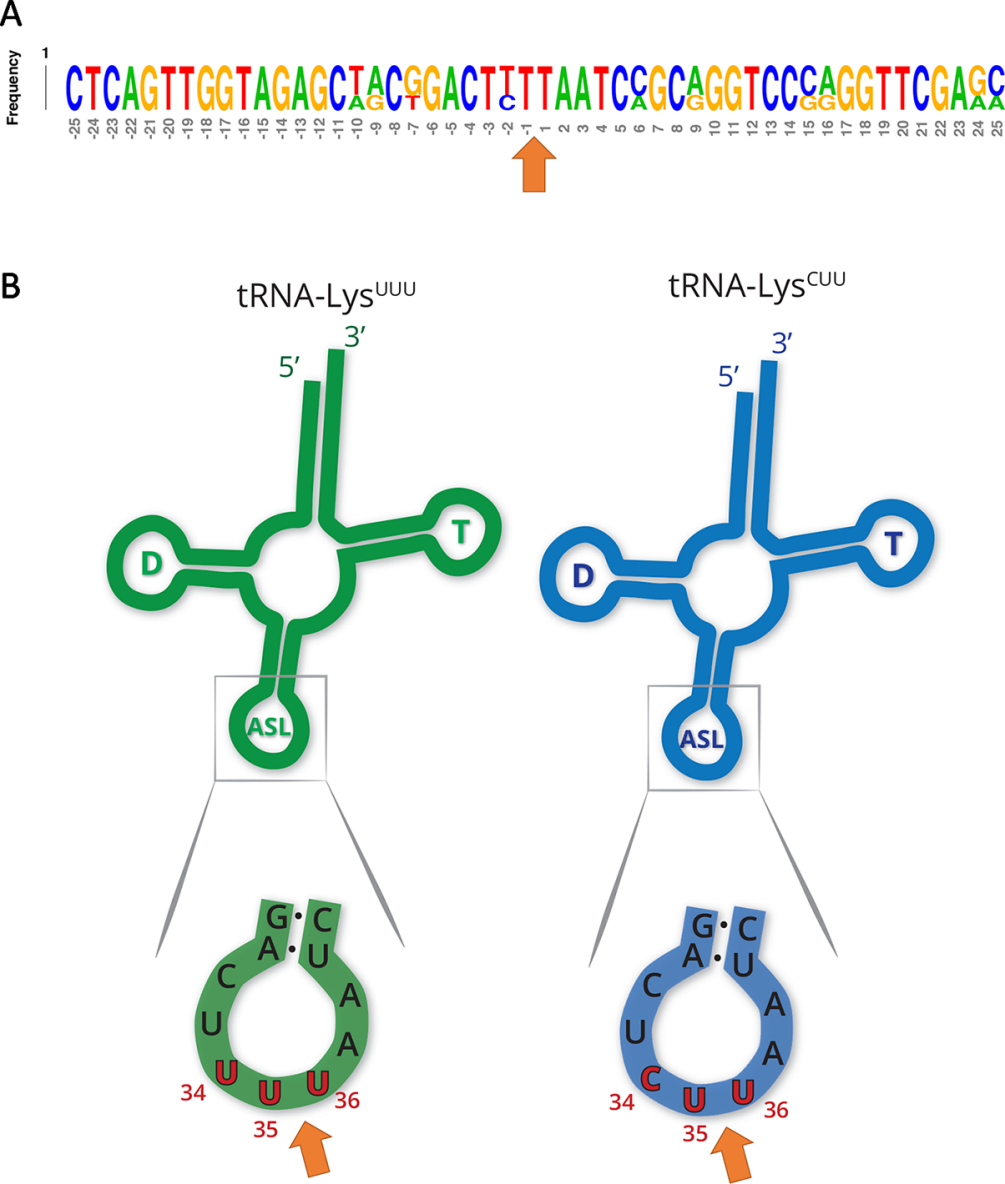
MazF-ms targets show high secondary structure and sequence similarity. **(A)** Frequency logo showing the DNA sequence similarity between tRNA^Lys-UUU^ and tRNA^Lys-CUU^ genes in the 50 nucleotides surrounding MazF-ms cleavage position (orange arrow). **(B)** Schematic representation of tRNA^Lys-UUU^ and tRNA^Lys-CUU^, illustrating the D-arm (D), T-arm (T) and anticodon stem loop (ASL) portions of the tRNA. ASL is partially shown in greater detail to emphasize the sequence and secondary structure near the cleavage site (orange arrow). Numbering in red indicates the nucleotide position of the anticodon in the mature tRNA molecule.

Other tRNA-cleaving toxins, such as MazF-mt9 and VapC-mt11 (Schifano et al., 2016; Cintrón et al., 2019), rely on both sequence and secondary structure to accurately recognize their targets. Accordingly, the two tRNA^Lys^ identified here as MazF-ms targets only differ by one nucleotide in the ± 5 nt region surrounding the cleavage site (**Figure 3A**). This site is located in the anticodon stem loop, where the predicted secondary structure is highly conserved. More specifically, cleavage occurred between the second and third bases of the anticodons (UU↓U and CU↓U, **Figure 3B**), presumably inactivating these tRNAs.

### 5’ RNA-Seq of MazF-ms-Expressing Cells Reveals Ribosome Stalling

**Figure 4:**
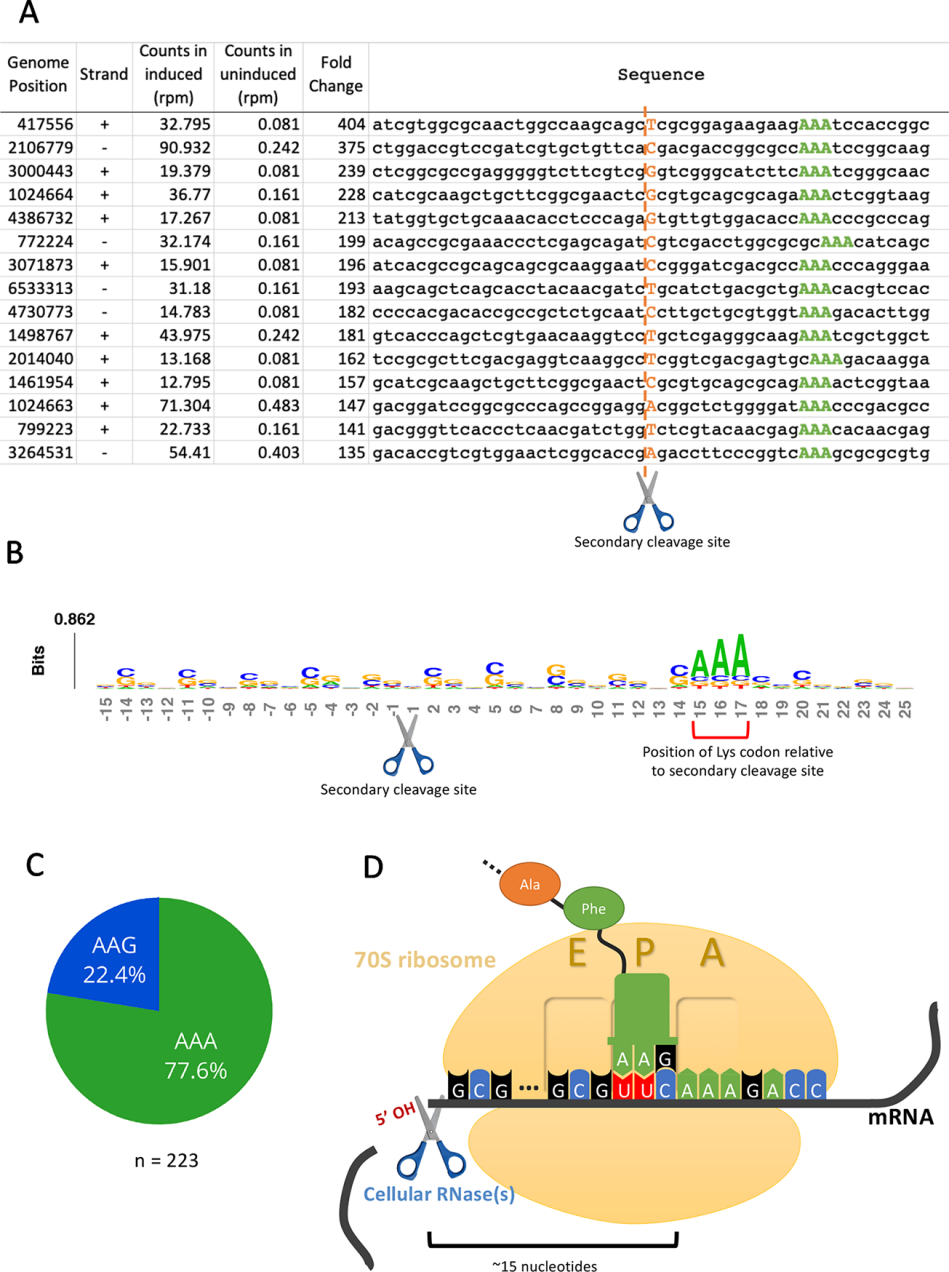
5’RNA-seq serendipitously reveals ribosome stalling at lysine codons. **(A)** Top mRNA hits found in the 5’RNA-seq dataset. The 50 nucleotides surrounding the secondary cleavage site (dotted line) generated by one or more cellular RNases (scissor) are shown. Lysine AAA codons are indicated in green, approximately 15 nucleotides downstream of the cleavage site. The first nucleotide (adjacent to the 5’ OH) of the read is highlighted in orange. Counts are normalized to reads per million (rpm). **(B)** Sequence logo summarizing the 130 detected mRNA hits. The height of each nucleotide is proportional to its frequency at that given position. Positions are numbered relative to the secondary RNase cut site (scissor). Red bracket indicates the position of lysine AAA codons. **(C)** Proportion of stalling in AAA codons vs. AAG codons found by 5’ RNA-seq in 223 annotated transcripts with at least 1 rpm in the induced sample and containing a lysine codon at or near +15. **(D)** Schematic representation demonstrating the events following the depletion of the cellular levels of tRNA^Lys^ by MazF-ms. Due to the lack of available tRNA^Lys^, translating ribosomes stall mainly at lysine AAA codons at the A site. Ribosome stalling events likely trigger mRNA cleavage at 5’ side of the stalled ribosome by one or more cellular RNases (scissor).

Having established that the primary targets were tRNA^Lys-UUU^ and tRNA^Lys-CUU^, we questioned whether the depletion of these tRNAs would lead to ribosome stalling in *M. smegmatis* at the mRNA codons requiring these tRNAs as we had previously observed for *M. tuberculosis* (Barth et al., 2019). Indeed, we identified 130 cleaved mRNAs in our 5’ RNA seq dataset that were not similar in sequence to the tRNA targets at the cleavage site and are not expected to have the secondary structure requirements for MazF-ms recognition demonstrated by Schifano et al. (2016). When aligned by their 5’ ends, these transcripts showed a clear AAA or AAG consensus sequence approximately 15 nt downstream of the 5’ OH end (**Figures 4A, B**), the cognate Lys codons for tRNA^Lys-UUU^ and tRNA^Lys-CUU^, respectively. As we had recently proven by Ribo-seq, the 15-nt spacing from the codon to the 5’ OH end indicates that a stalled ribosome was bound at this position of the mRNA *in vivo* (Barth et al., 2019). The 15 nts represents the approximate distance from the 5’ side of the translating ribosome to the A-site (**Figures 4D**, Barth et al., 2019). Thus, evidence of the stalled ribosome is fortuitously revealed by 5’ RNA-seq because upon stalling, the mRNA is then cleaved on its 5’ side of the ribosome by one or more cellular RNase(s)—not MazF-ms—that generates a 5’ OH upon cleavage.

We have described the same cascade of events for just one of the 11 MazF family members in *M. tuberculosis*, MazF-mt9, in which tRNA^Lys43-UUU^ depletion leads to ribosome stalling and cleavage on the 5’ end of the ribosome. The observation of an analogous trend in the MazF-ms 5’ RNA-seq datasets indicating ribosome stalling and subsequent cleavage strongly suggests mechanistic conservation between MazF-mt9 and MazF-ms toxins regarding initial toxin-mediated tRNA cleavage followed by a secondary ribosome stalling/mRNA cleavage event. In *M. smegmatis*, however, although tRNA cleavage was significant for both isoacceptors, the vast majority of the observed stalled ribosomes (75%) paused at the rarer Lys AAA codon rather than the more frequent Lys AAG (**Figure 4C**). Therefore, as in *M. tuberculosis*, our data support a model in which MazF-ms acts by depleting the cellular pool of tRNA^Lys^ causing ribosome stalling at Lys codons (predominantly Lys AAA), followed by recruitment of another RNase that cleaves 5’ of the stalled ribosome (**Figure 4D**).

### MazF-ms Promotes Codon-Specific Translation

Next, we sought to characterize the proteomic changes promoted by the Lys AAA/AAG-specific ribosome stalling events mediated by MazF-ms. In order to distinguish proteins that were only synthesized after MazF-ms induction from preexisting “old” proteins, we adopted a method that utilizes a methionine mimetic called azidohomoalanine (AHA). AHA is incorporated into nascent peptides during translation, therefore only marking proteins that were synthesized after its addition to the media. Due to its azide moiety, proteins containing AHA residues can be captured using an alkyne-containing resin through a Cu(I)-catalyzed click reaction and then analyzed by quantitative mass spectrometry. We added AHA to the cultures after 4.5 h of MazF-ms induction, tagging only proteins that were newly translated.

**Figure 5:**
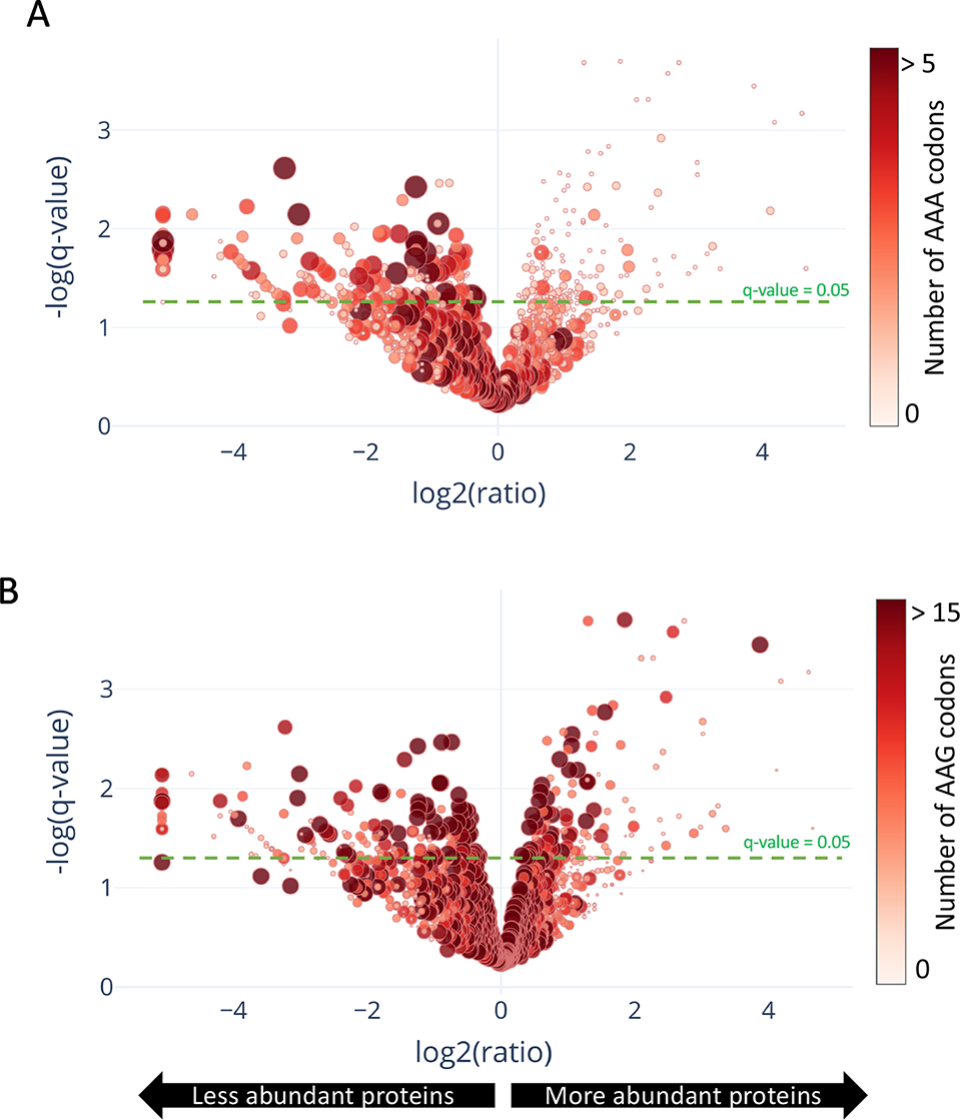
Translation of proteins rich in lysine AAA codons is reduced upon MazF-ms expression. **(A–B)** Volcano plot showing differentially translated proteins (circles) detected by quantitative mass spectrometry and their AAA **(A)** or AAG **(B)** codon content. *M. smegmatis* cultures expressing MazF-ms for 4.5 h were incubated with azidohomoalanine (AHA) to label only newly synthesized proteins after toxin expression. The color saturation and circle size are proportional to the number of AAA **(A)** or AAG **(B)** codons in the gene.

**Figure 6:**
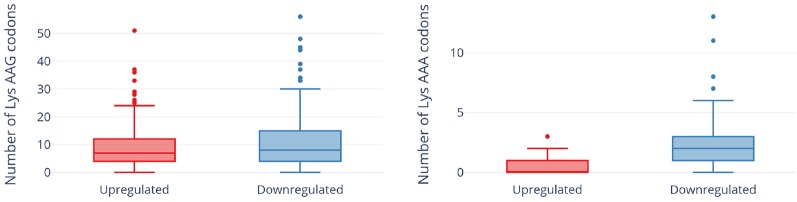
Expression of MazF-ms leads to global proteomic shifts based on the Lys AAA, but not Lys AAG, codon content. Distribution of AAG (left) or AAA (right) codons in significantly upregulated or downregulated proteins. Outliers are shown as individual dots.

In contrast to a translation shut-off model proposed for other tRNA-cleaving toxins (Winther et al., 2016), global translation was not halted. One hundred twenty six proteins were significantly more abundant in cells expressing MazF-ms compared to the controls (**Supplementary Table 1**, q-value < 0.05). Interestingly, a striking difference in AAA codon content was detected in these proteins compared to the less abundant ones (**Figure 5A**), i.e. upregulated proteins were generally Lys AAA codon deficient while downregulated proteins were generally Lys AAA codon rich. This trend was not as striking when the proteomics data was instead graphed based on AAG codon content (**Figure 5B**). These trends observed in newly synthesized proteins were concordant with the relative abundance of ribosome stalling events detected in our 5’ RNA-seq datasets at Lys AAA codons (>75% of transcripts with evidence of ribosome stalling). Finally, the ability of MazF-ms to preferentially influence the overall cellular pool of Lys AAA codon-containing proteins over those containing Lys AAG codons is also graphed in **Figure 6**.

### MazF-ms Reduces Translation of Critical Components of the DNA Replication Machinery While Concomitantly Supporting Synthesis of Stress Response Proteins

**Figure 7:**
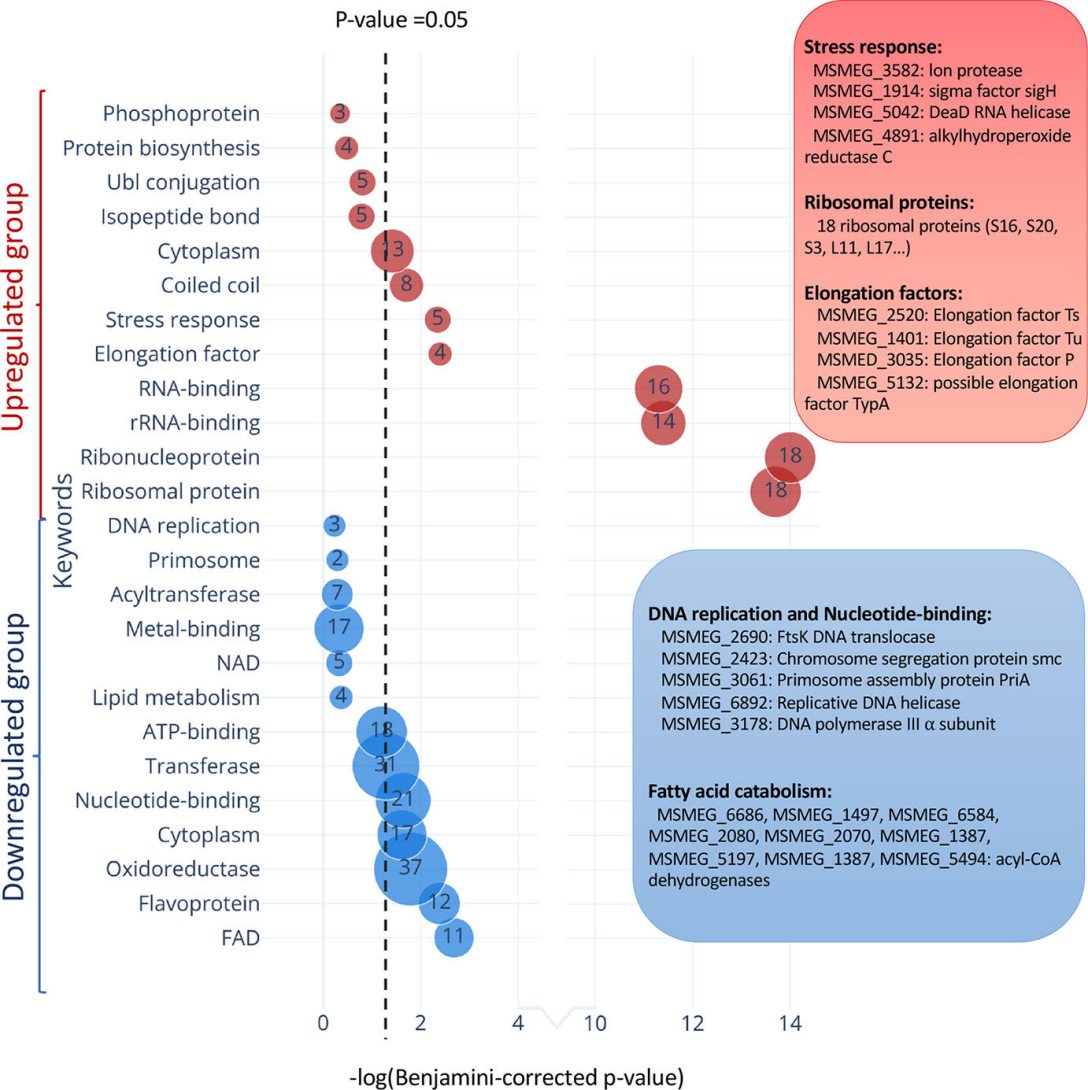
Functional classification of differentially translated proteins during MazF-ms expression. Enriched DAVID UP_KEYWORDS (corresponding to UniProt keywords) were generated by analyzing the upregulated (red) or downregulated (blue) proteins identified in azidohomoalanine (AHA)-proteomics. The diameter of each circle and its numbering correspond to the number of genes associated with the given keyword. Examples of genes in the main groups are described by relevant categories in the corresponding color-matched boxes.

In TA systems, specific stresses trigger degradation of the cognate antitoxin by a protease, freeing toxin to act within the cell (Yamaguchi et al., 2011; Harms et al., 2018; Song and Wood, 2018). Therefore, overexpression of MazF-ms (used in this analysis and throughout this work) is intended to mimic natural toxin activation that occurs when cells are exposed to the relevant stress. After observing hundreds of differentially translated proteins in **Figure 5**, we analyzed the two datasets comprising the more abundant or less abundant proteins following MazF-ms toxin expression using the Function Annotation Tool in the Database for Annotation, Visualization and Integrated Discovery (DAVID) platform (Huang et al., 2009a; Huang et al., 2009b). This tool within DAVID was used to identify functionally similar proteins which were enriched in the two datasets (**Figure 7**). Since toxin overexpression simulates toxin activation by exposure to stress, the proteins identified using DAVID are expected to be physiologically relevant.

Among the 171 statistically significant downregulated proteins from **Figure 5A** (in blue in **Figure 7**), there were some notable trends consistent with cells in a state of growth arrest (**Supplementary Table 2**). First, there were 11 downregulated enzymes involved in “fatty acid catabolism” (FAD heading; blue box **Figure 7**). Fatty acid catabolism occurs predominantly through successive rounds of β-oxidation, a process whereby even-chain fatty acids are degraded to acetyl-CoA and odd-chain fatty acids are degraded to acetyl-CoA and propionyl-CoA. Second, there were five functionally related proteins within the “DNA replication” and “nucleotide binding” categories (blue box **Figure 7**). In the DNA replication category, one critical protein was downregulated, the only catalytic subunit of the three subunit core DNA polymerase III enzyme (α subunit, MSMEG_3178). This core enzyme is a component of the DNA polymerase III holoenzyme that mediates DNA replication in bacteria. Within the nucleotide binding group were a spectrum of proteins whose downregulation is also logical for cells in a state of growth arrest: RecA (MSMEG_2723) is involved in DNA repair, the DNA translocase FtsK (MSMEG_2690) is localized at the septum where cell division occurs, chromosome segregation protein SMC (MSMEG_2423) and the priA (MSMEG_3061) are components of the primosome protein complex that activates DNA replication forks.

There were 126 upregulated proteins upon MazF-ms expression. When this dataset was subjected to the Functional Annotation Tool in DAVID, there were several proteins whose upregulation were also consistent with the growth arrested state of MazF-ms expressing cells. Four proteins were in the “stress response” category: Lon protease (MSMEG_3582), an ATP-dependent RNA helicase DEAD/DEAH box family protein (MSMEG_5042), sigma factor SigH (MSMEG_1914) and alkylhydroperoxide reductase (MSMEG_4891). Lon is a stress-responsive protease. Since it is known to degrade all TA system antitoxins in *Escherichia coli* (Gerdes and Maisonneuve, 2012), it may also have an analogous role and cleave the MazE-ms antitoxin in *M. smegmatis*. Uniprot places the MSMEG_5042 RNA helicase as functioning in ribosome biogenesis, mRNA degradation and translation initiation. The alternate sigma factor SigH is activated by oxidative, heat and nitric oxide stress (Sharp et al., 2016) while Uniprot places alkylhydroperoxide reductase MSMEG_4891 in protection from oxidative damage by detoxifying peroxides.

Consistent with the sustained protein synthesis while MazF-ms was being expressed, we observed new synthesis of a subset of elongation factors and ribosomal proteins. Four elongation factors were upregulated: EF-Ts (MSMEG_2520), EF-Tu (MSMEG_1401), EF-P (MSMEG_3035) and BipA/TypA ribosome-binding GTPase (MSMEG_5132). Many of these elongation factors are associated with the bacterial stress response. Finally, 18 of the 51 ribosomal proteins were also upregulated. The other 33 ribosomal protein levels were stable.

## Discussion

Bacterial genomes are under constant pressure to remain compact while also retaining genes that provide a competitive edge for survival in their natural environments. Acquisition of TA systems in bacterial genomes is thought to represent one potent vehicle for stress protection. In contrast to the ~90 TA systems in its pathogenic relative *M. tuberculosis*, the *M. smegmatis* genome harbors just three TA systems (MazEF, PhD-Doc, and VapBC) as one facet of its stress survival armamentarium. A thorough understanding on how a TA system acts to protect its host from stress requires determination of the function of the toxin, and the function of the toxin is informed by determining its intracellular target. Since all MazF toxins are generally single-strand and sequence-specific endoribonucleases, in this work we identified the RNA target of the MazF-ms toxin using 5’ RNA-seq which revealed its detailed mechanism of action. To our surprise, MazF-ms did not behave like the vast majority of MazF toxins that appear to predominantly cleave mRNAs (reviewed in Masuda and Inouye, 2017). Instead, MazF-ms behaves almost exactly like the only other known exception, MazF-mt9, one of the 11 MazF family members in *M. tuberculosis* (Barth et al., 2019).

**Figure 8:**
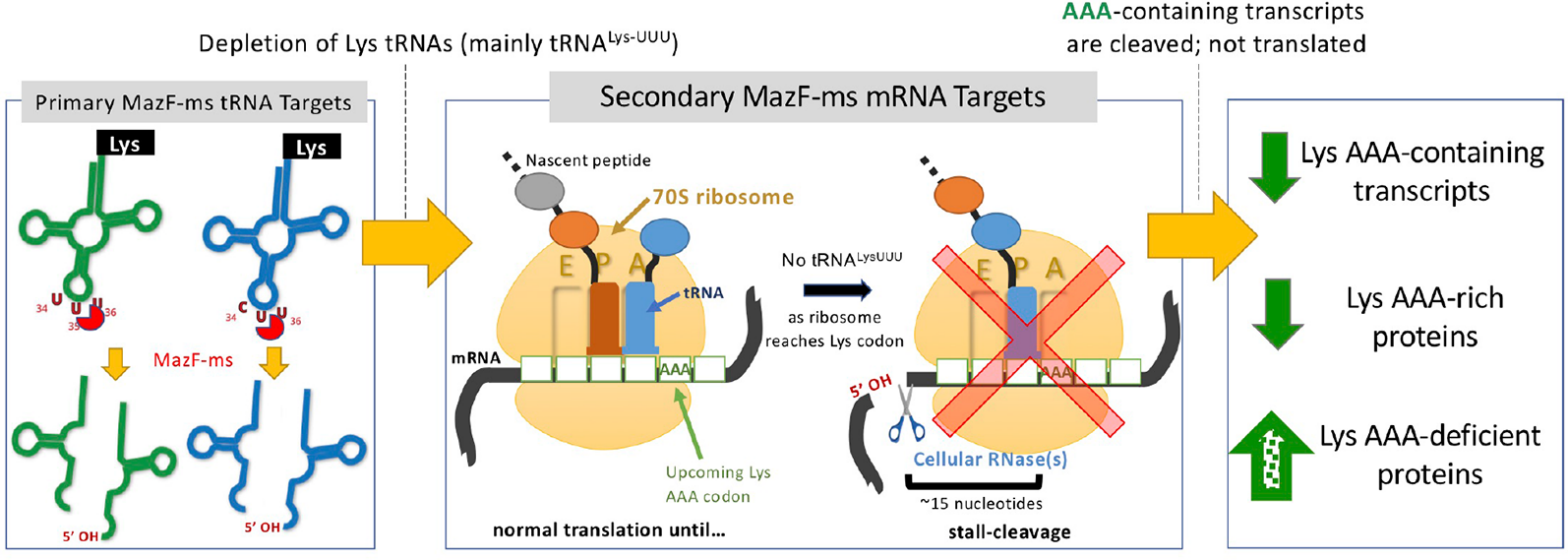
Summary of the proposed MazF-ms mechanism of action. MazF-ms primarily targets tRNA^Lys-UUU^ and tRNA^Lys-CUU^, depleting their intracellular levels. Inactivation of these tRNAs lead to selective ribosome stalling predominantly in rare Lys-AAA codons, culminating in cleavage of the transcript. This results in codon-biased proteomic changes, favoring Lys-AAA-depleted transcripts.

MazF-mt9 is an outlier because it requires both structure and sequence for its highly specific recognition of a single tRNA isoacceptor. This requirement for structure is much like VapC toxins, even though MazF-mt9 and VapC toxins lack sequence or structural similarity (Cruz et al., 2015; Schifano et al., 2016; Winther et al., 2016; Schifano and Woychik, 2017; Cintrón et al., 2019). MazF-ms now represents the second example of a MazF toxin that targets tRNA for cleavage, thus reducing the levels of only this tRNA species *in vivo*. Since we were able to unequivocally detect MazF-ms target RNAs with the required 5’-OH using 5’ RNA-seq, there was no apparent masking of the precise cleavage site due to the presence of an RNA modification. This surgical depletion of just one tRNA results in ribosome stalling at codons requiring this depleted tRNA and proteome remodeling to favor sustained synthesis of only AAA-deficient proteins (**Figure 8**).

Why is Lys tRNA such an important conduit for proteome remodeling, ostensibly during stress, in mycobacteria? It appears that many proteins critical for stress survival in these GC rich genomes (67.4% GC in *M. smegmatis;* 65.6% in *M. tuberculosis*) are deficient in AAA Lys codons, allowing for their sustained translation while the cell can save energy by not synthesizing proteins that do not contribute to this endpoint. Indeed, as discussed above, many of the upregulated proteins annotated in the stress response category have direct roles in one or more stress responses: cold shock, heat shock, oxidative stress and nitric oxide stress. The stress-specific sigma factor SigH was also upregulated. Therefore, it is implicated as the primary RNA polymerase sigma factor enlisted for the sustained transcription of *M. smegmatis* genes whose proteins were upregulated after toxin expression. However, other upregulated proteins in our dataset that are not commonly associated with the stress response, i.e. at least some elongation factors, appear to have indirect roles (reviewed in Starosta et al., 2014). EF-Tu is maximally expressed during stress in *E. coli* (Muela et al., 2008). Since the guanine nucleotide exchange factor EF-Ts assembles with EF-Tu in a 2:2 stoichiometry (Kawashima et al., 1996), it is expected to be expressed at levels equivalent to EF-Tu. EF-P rescues ribosomes stalled at poly-proline stretches. However, EF-P is only active when lysinylated or hydrozylysinylated (Doerfel et al., 2013; Ude et al., 2013). Thus, the lysine from cleaved Lys tRNAs might be recycled and used to activate EF-P. Finally, BipA/TypA, while not essential, appears to confer a growth advantage by regulating the synthesis of a subclass of proteins in cells under cold shock, low pH, oxidative stress, antimicrobial peptide stress and detergent stress.

## Data Availability Statement

The proteome data and RNA-seq data have been deposited and made public in the Massive database under the accession number MSV000084300 (https://massive.ucsd.edu/ProteoSAFe/dataset.jsp?task=1c14b9a1fd354c8fbb7ddcf03ace9fdd) and SRA under the accession number PRJNA564437 (https://www.ncbi.nlm.nih.gov/bioproject/PRJNA564437), respectively.

## Author Contributions

VB performed all experiments. Data analysis/interpretation was performed by VB. NW directed the project and acquired funding. NW and VB wrote the manuscript.

## Conflict of Interest

The authors declare that the research was conducted in the absence of any commercial or financial relationships that could be construed as a potential conflict of interest.
