# Supplementary material for: The Sole Mycobacterium smegmatis MazF Toxin Targets tRNALys to Impart Highly Selective, Codon-Dependent Proteome Reprogramming
Source: Front Genet. 2020 Feb 14;10:1356. doi: 10.3389/fgene.2019.01356 (PMC7033543; doi:10.3389/fgene.2019.01356)
Supplement: Supplementary file 1 [file Image_1.pdf]

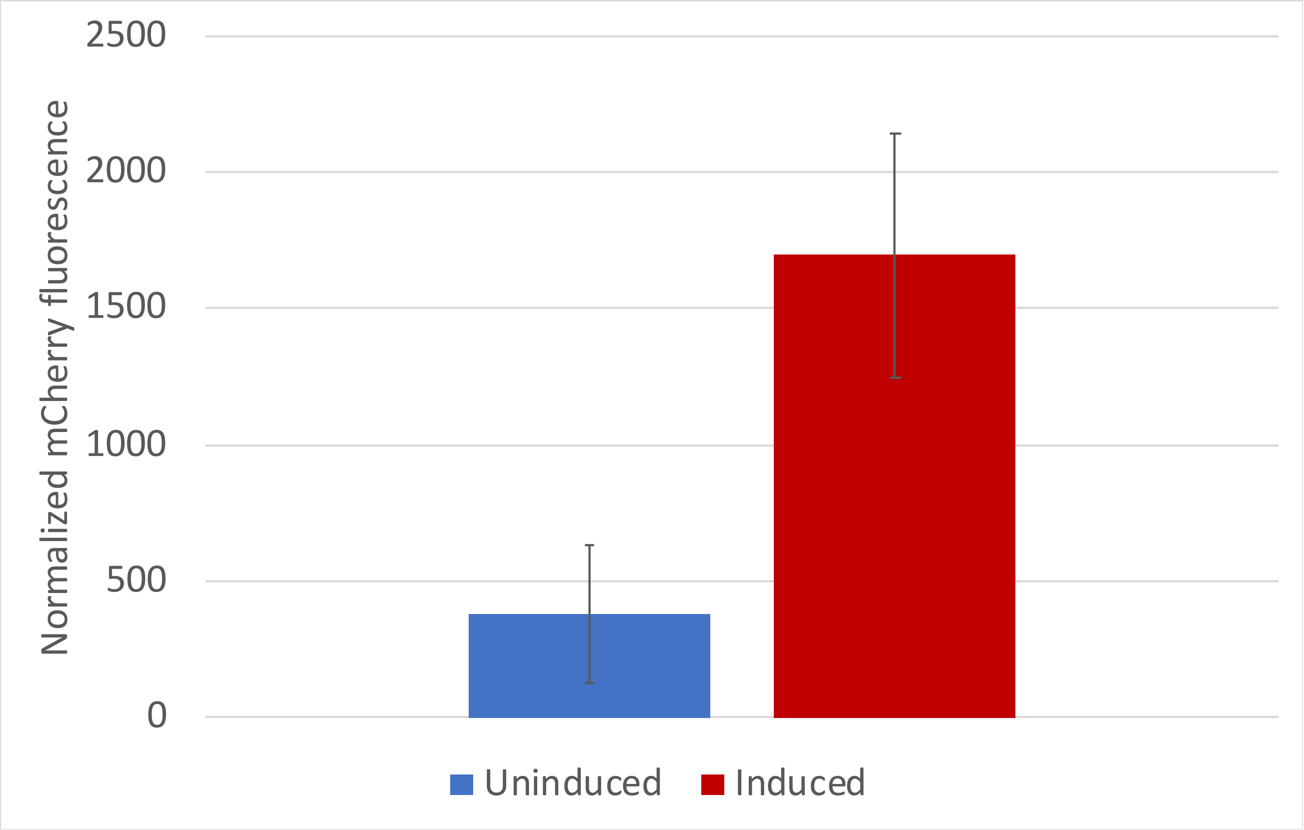

Supplementary Figure 1. Expression levels of pMC1s vector in *M. smegmatis*. pMC1s vector expression was estimated by cloning a fluorescent reporter gene (mCherry) and inducing its expression with 200 ng/ml of anhydrotetracycline for approximately 6h. mCherry fluorescence was measured (Excitation: 585 nm, Emission: 610 nm) in a Synergy HT 96-well plate spectrophotometer. Raw fluorescence reads were normalized by OD and by auto-fluorescence of a culture that did not contain mCherry.
